# A new intelligently optimized model reference adaptive controller using GA and WOA-based MPPT techniques for photovoltaic systems

**DOI:** 10.1038/s41598-024-57610-0

**Published:** 2024-03-21

**Authors:** Nassir Deghfel, Abd Essalam Badoud, Farid Merahi, Mohit Bajaj, Ievgen Zaitsev

**Affiliations:** 1 Setif Automatic Laboratory, Electrical Engineering Department, Ferhat Abbas University Setif 1, Setif, Algeria; 2 Department of Electrical Engineering, Graphic Era (Deemed to be University), Dehradun, 248002 India; 3 Hourani Center for Applied Scientific Research, Al-Ahliyya Amman University, Amman, Jordan; 4 Graphic Era Hill University, Dehradun, 248002 India; 5 Applied Science Research Center, Applied Science Private University, Amman, 11937 Jordan; 6 Department of Theoretical Electrical Engineering and Diagnostics of Electrical Equipment, Institute of Electrodynamics, National Academy of Sciences of Ukraine, Peremogy, 56, Kyiv-57, 03680 Ukraine

**Keywords:** Maximum power point tracking, Photovoltaic systems, Model reference adaptive control, Adaptive neuro-fuzzy inference system, Genetic algorithm, Renewable energy, Convergence analysis, Energy science and technology, Engineering, Mathematics and computing

## Abstract

Recently, the integration of renewable energy sources, specifically photovoltaic (PV) systems, into power networks has grown in significance for sustainable energy generation. Researchers have investigated different control algorithms for maximum power point tracking (MPPT) to enhance the efficiency of PV systems. This article presents an innovative method to address the problem of maximum power point tracking in photovoltaic systems amidst swiftly changing weather conditions. MPPT techniques supply maximum power to the load during irradiance fluctuations and ambient temperatures. A novel optimal model reference adaptive controller is developed and designed based on the MIT rule to seek global maximum power without ripples rapidly. The suggested controller is also optimized through two popular meta-heuristic algorithms: The genetic algorithm (GA) and the whale optimization algorithm (WOA). These meta-heuristic approaches have been exploited to overcome the difficulty of selecting the adaptation gain of the MRAC controller. The reference voltage for MPPT is generated in the study through an adaptive neuro-fuzzy inference system. The suggested controller’s performance is tested via MATLAB/Simulink software under varying temperature and radiation circumstances. Simulation is carried out using a Soltech 1sth-215-p module coupled to a boost converter, which powers a resistive load. Furthermore, to emphasize the recommended algorithm’s performance, a comparative study was done between the optimal MRAC using GA and WOA and the conventional incremental conductance (INC) method.

## Introduction

Among the most disturbing topics in the status quo is the spread of environmental pollution worldwide^1^. The primary factor that causes ecological pollution is fossil fuels because of their use in energy production and industrial fields^2,3^. No doubt, finding a new energy source has become necessary to reduce the use of fossil fuels and their emissions^4,5^. Among the robust proposed solutions is renewable energy, like solar and wind energy. The demand for renewable energy as an alternative source is growing^6,7^. Solar energy is one of the most popular sources because it is limitless and spotless to produce energy without harmful emissions^8,9^. Depending on the photovoltaic (PV) effect, a solar cell converts the irradiation into electrical energy through many physical processes^10^. Even though all the PV cells have enticing features, their energy conversion efficiency is still relatively low^11^. Figure 7 shows the power-voltage (P–V) diagram for a PV array, demonstrating the variation of the PV power concerning PV voltage under various amounts of temperature and irradiance. It is clear that only under steady-state environmental conditions does the PV cell provide the highest operating point, called the maximum power point (MPP)^9,12^^,^^13^. To achieve this point, we must employ a critical device known as a maximum power point tracking (MPPT) controller^14^. Today, researchers worldwide seek to develop and create new methods to extract as much power from PV panels^15,16^. In the literature^8,17^^,^^18^, we can find various classifications of these MPPT techniques.

Among the classical MPPT techniques, we mention Perturb and observe P&O^19–21^, Incremental Conductance (INC)^22–24^, and Hill Climbing (HC)^25–27^. However, each technique has specific strengths and weaknesses; for example, P&O and HC are simple and require fewer sensors than other techniques^28,29^. In^30^, a modified perturbation and observation have been proposed to avoid the drift due to the traditional P&O’s incorrect choice. The INC algorithm was introduced to resolve P&O issues. In^31^, an improved MPPT strategy based on INC is suggested to increase productivity and efficiency under fast-changing irradiance. Ref^32^, has been proposed an improved P&O using simulated annealing (SA) algorithm, the proposed algorithm exhibits better performance in varying weather conditions and partial shading Nevertheless, the classical MPPT methods and even those developed are still weak under varying environmental conditions and rapid oscillations around the maximum power.

In terms of dynamic weather-varying conditions, intelligent MPPT techniques^33^ are often used, including fuzzy logic control (FLC)^34,35^, artificial neural networks (ANN)^36–38^, adaptive neuro-fuzzy inference systems (ANFIS)^39^, sliding mode control^40^, and Gauss–Newton method-based MPPT^34^. These techniques are very efficient and faster for tracking the MPP. Nevertheless, they require many data sets to train and improve tracking accuracy, especially ANN and ANFIS-based MPPT; their implementation can be challenging.

Recently, bio-inspired algorithms like Particle Swarm Optimization (PSO)^41^, Genetic Algorithm (GA)^42^, Grey Wolf Optimization (GWO)^43^, and Whale Optimization Algorithm^44^ have been widely used in PV systems to determine the maximum power point, especially under partial shading conditions^45^. In^46^ a new MPPT called the slime mold golden sine algorithm was proposed in order to address the partial shading problem, the proposed SMGSA method exhibits superior effectiveness and enhancement. Most of these strategies follow an identical sequence or procedure to accomplish optimization^47,48^. Although bio-inspired solutions can effectively handle the challenge of partial shading, their efficacy depends entirely on the parameters chosen and the starting conditions^49^.

Combining bio-inspired algorithms with other MPPT approaches can overcome this restriction in bio-inspired algorithms. In^50^, an invented P&O with adaptive variable step size based on a PID controller optimized using the Genetic Algorithm was introduced. It shows less oscillation around the MPP and improves efficiency. Ref.^51^ has suggested a fuzzy logic controller optimized through the cuckoo strategy optimization approach (COA-FLC) for Maximum Power Point Tracking (MPPT) under various meteorological conditions. This COA-FLC has improved the convergence time and minimized the output ripple power. A fractional order based MPPT enhanced using different metaheuristic techniques was suggested in^52^, The results obtained demonstrated a high level of accuracy and increased robustness.

To avoid such improbable and substantial variations during the transitory phase, researchers proposed a two-level MPPT control architecture^53^. In^54^, the ripple correlation control algorithm was the initial loop of control, while the model reference adaptive controller MRAC was the second. The separation of these control algorithms results in the attainment of MPPT while ensuring the entire system’s stability. Another solution is described in^55^; an improved model reference adaptive controller was associated with incremental conductance. A set of tests and simulations using PSIM and experimental tests are employed to verify the effectiveness of the suggested methodology. However, most plants have PV systems and boost converters, mathematically modeled as second-order systems. Indeed, the performance of traditional MRAC tracking systems could be better for second-order systems. To improve MPPT performance, a novel MRAC structure has been expanded from the first to the second order^56^.

**Figure 1:**
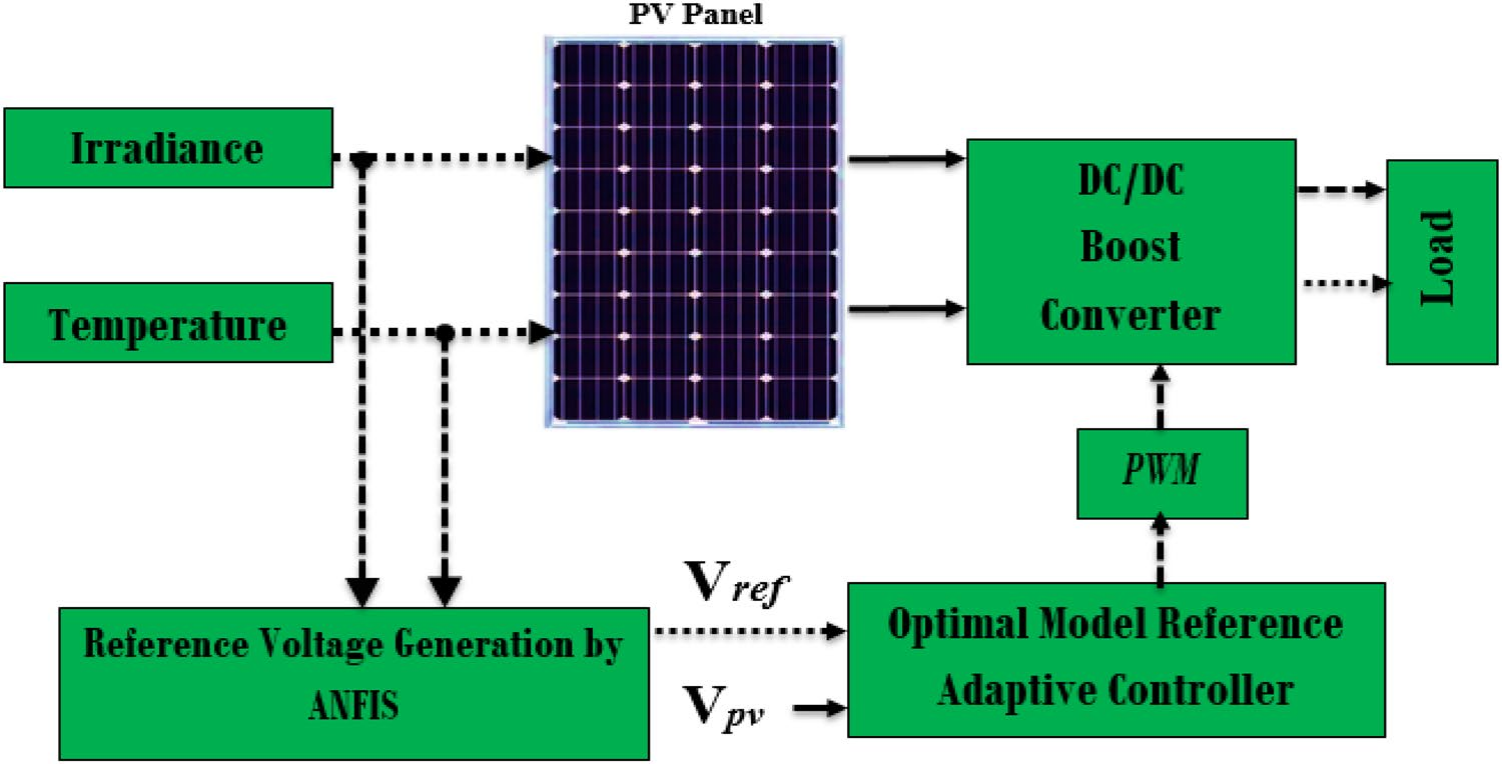
The proposed MPPT block diagram for the PV system.

Nevertheless, one of the significant issues caused by non-linearity is determining MRAC adaptation gains according to specific methods. As described in references^57,58^, the authors investigated the influence of changing the adaptation gain on the overall system’s performance, including the time response and the oscillation in the response. In this article, meta-heuristic algorithms have been used to overcome this difficulty. Two optimization techniques are applied to tune the parameters of MRAC and improve its dynamic performance, namely the genetic algorithm (GA) and the whale optimization algorithm (WOA)^59^. In addition, an adaptable Neuro-Fuzzy Inference System (ANFIS) has been employed to produce voltage references for maximum power capacity, which a designed controller subsequently tracks; Fig. 1 illustrates the block diagram of the developed system.

The rest of this paper is organized as follows: Section “Mathematical modeling of photovoltaic array” describes a photovoltaic array's mathematical modeling and the boost converter’s mathematical representation. The neuro-fuzzy network and the proposed optimal model reference adaptive controller, OMRAC, are presented in Section “Dynamics modeling of the DC–DC boost converter”. Section “Proposed MPPT” contains the simulation results, discussion, conclusion, and future work.

## Mathematical modeling of photovoltaic array

### Single diode model

**Figure 2:**
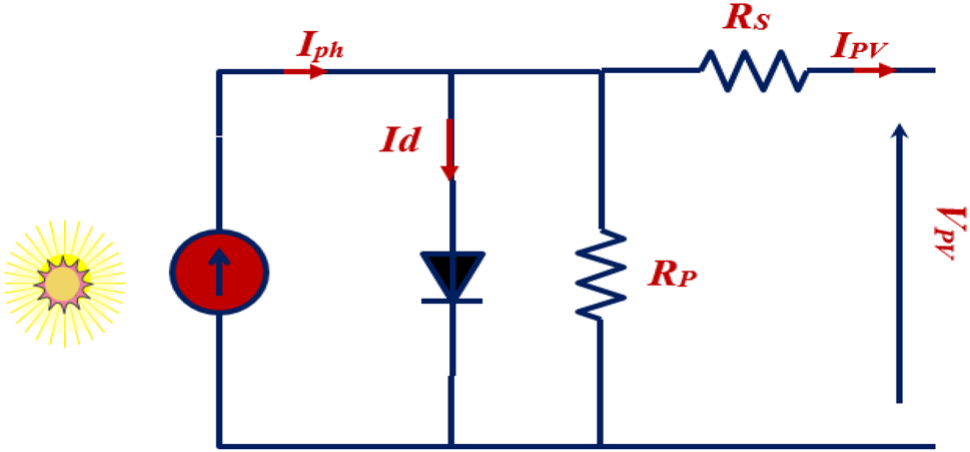
PV cell circuit using a single diode model.

Several mathematical models illustrating solar panels' operation and performance are documented in the literature. Indeed, real-time simulation requires equivalent circuit modeling of PV cells. The most popular approximate equivalent model researchers use is the single-diode model, as presented in Fig. 2. The used circuit consists of at least four components: a current source *I*_*Ph*_*,* a diode D, a parallel resistor *R*_*p*_, and a series resistor *R*_*s*_.

According to the single diode equivalent model of the PV cell presented in Fig. 2, the output current* I*_*PV*_ can be described as follows^60^.

1 $${{\text{I}}}_{{\text{PV}}}={{\text{N}}}_{{\text{p}}}{{\text{I}}}_{{\text{ph}}}-{{\text{N}}}_{{\text{p}}}{{\text{I}}}_{\mathbf{r}\mathbf{s}}.\left[{\text{exp}}\left(\frac{{\text{q}}\left({\text{v}}+{{\text{R}}}_{{\text{s}}}{{\text{I}}}_{{\text{PV}}}\right)}{{{\text{AkTN}}}_{{\text{s}}}}\right)-1\right]-{{\text{N}}}_{{\text{p}}}\left[{\text{q}}\left(\frac{\left({\text{v}}+{{\text{R}}}_{{\text{s}}}{{\text{I}}}_{{\text{PV}}}\right)}{{{\text{N}}}_{{\text{s}}}{{\text{R}}}_{{\text{sh}}}}\right)\right],$$

The reverse saturation current of the cell is referred to as *I*_*rs*_. V denotes the voltage of the cell, whereas *N*_*s*_ indicates the number of PV cells linked in series at the same time. *N*_*p*_ indicates the number of photovoltaic cells linked in parallel. *K* represents the Boltzmann constant, q symbolizes an electron’s charge, *T* denotes Kelvin’s temperature, and *A* represents the diode ideality constant. Based on Eq. (2), the irradiance of the sun *E* and the ambient temperature *T* are two of the main factors determining the *I*_*Ph*_.

2 $${{\text{I}}}_{{\text{ph}}}=\left[{{\text{I}}}_{{\text{sc}}}+{{\text{k}}}_{{\text{i}}}\left(\mathbf{T}-{{\text{T}}}_{{\text{r}}}\right)\right]\left(\frac{{\text{E}}}{1000}\right),$$

*K*_*i*_ represents the short-circuit current, *T* denotes the temperature coefficient of the cell, *I*_*sc*_ is the short-circuit current, and *E* represents the variation in solar radiation. Equation (3) gives the saturation current in cell *I*_*rs*_*.* A strong correlation exists between temperature and saturation current^61,62^.

3 $${{\text{I}}}_{{\text{rs}}}={{\text{I}}}_{{\text{rr}}}{\left[\frac{{\text{T}}}{{{\text{T}}}_{{\text{r}}}}\right]}^{3}{\text{exp}}\left(\left[\frac{{{\text{qE}}}_{{\text{G}}}}{{\text{k}}.{\text{A}}}\right]\left[\frac{1}{{{\text{T}}}_{{\text{r}}}}-\frac{1}{{\text{T}}}\right]\right),$$

*I*_*rr*_: represents the reverse saturation corresponding to *T*_*r*_; *T*_*r*_: is the cell reference temperature; *E*_*G*_: is the band-gap energy of the semiconductor used in the cell.

### Triple diode model

**Figure 3:**
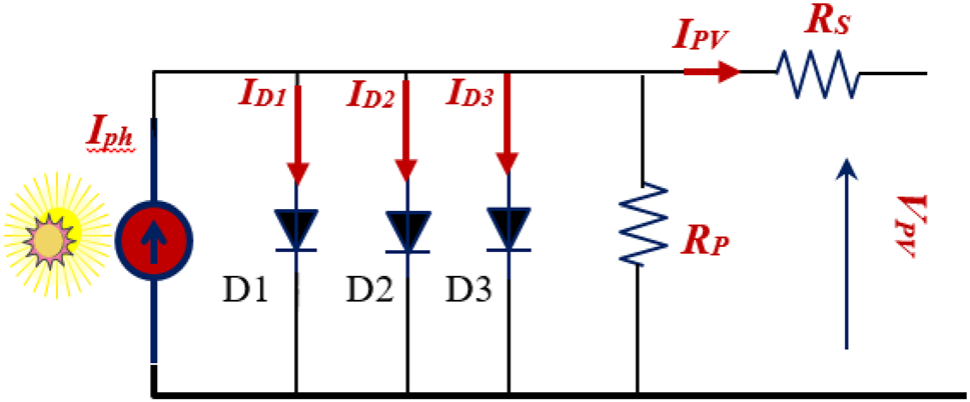
PV cell circuit using triple diode model.

The PV cell scheme based triple diode model is illustrated in Fig. 3. This model taken into consideration two additional diodes as in the single diode model^63,64^. The expression of the output current can be given as following^65^.

4 $${{\text{I}}}_{{\text{PV}}}={{\text{N}}}_{{\text{p}}}{{{\text{I}}}_{{\text{ph}}}-{\text{N}}}_{{\text{p}}}{{\text{I}}}_{01}\left({{\text{e}}}^{\frac{{\text{q}}({\text{v}}+{{\text{R}}}_{{\text{s}}}{{\text{I}}}_{{\text{PV}}}}{{{\text{AKTN}}}_{{\text{s}}}{{\text{n}}}_{1}}}-1\right)-{{\text{N}}}_{{\text{p}}}{{\text{I}}}_{02}\left({{\text{e}}}^{\frac{{\text{q}}({\text{v}}+{{\text{R}}}_{{\text{s}}}{{\text{I}}}_{{\text{PV}}}}{{{\text{AKTN}}}_{{\text{s}}}{{\text{n}}}_{2}}}-1\right) -{{\text{N}}}_{{\text{p}}}{{\text{I}}}_{03}\left({{\text{e}}}^{\frac{{\text{q}}({\text{v}}+{{\text{R}}}_{{\text{s}}}{{\text{I}}}_{{\text{PV}}}}{{{\text{AKTN}}}_{{\text{s}}}{{\text{n}}}_{3}}}-1\right)\left({{\text{N}}}_{{\text{p}}}\frac{{\text{q}}({\text{v}}+{{\text{R}}}_{{\text{s}}}{{\text{I}}}_{{\text{PV}}}}{{{\text{N}}}_{{\text{s}}}{{\text{R}}}_{{\text{sh}}}}\right)$$

where $I_{01} ,I_{02}$ and $I_{03}$ denoted to the reverse saturation current of each diode.

## Dynamics modeling of the DC-DC boost converter

A traditional MPPT algorithm relies on Eq. (4) to determine the converter's duty cycle when the system is stable. On the other hand, the MPPT controller needs to consider the dynamics related to the duty cycle *d* and the PV voltage *V*_*PV*_ to optimize transient responses^66,67^. When the duty cycle is adjusted to reflect varying environmental circumstances, the MPPT controller should remove any transient fluctuations in the PV voltage^68–70^.

4 $${{\text{V}}}_{{\text{PV}}}={\left(1-{\text{d}}\right)}^{2}\left({{\text{R}}}_{0}{{\text{I}}}_{{\text{PV}}}\right)$$

**Figure 4:**
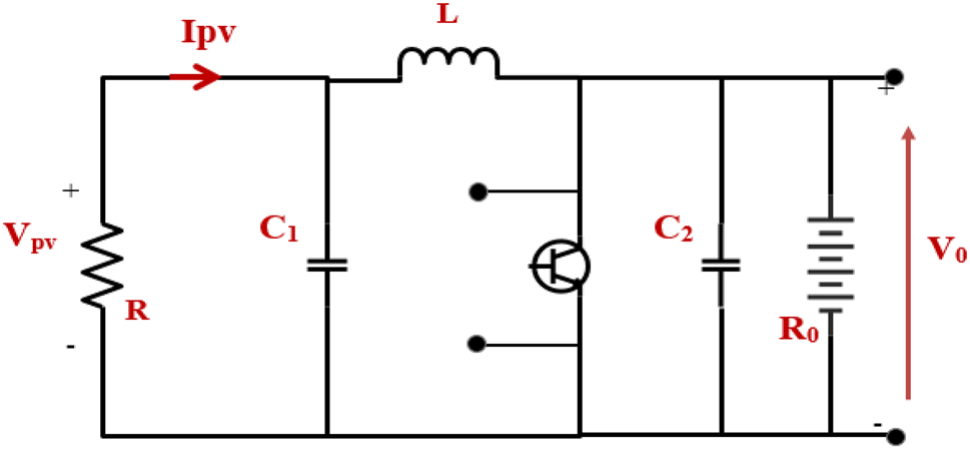
PV small signal model coupled to the boost converter.

As suggested in^71^,^72^, a small signal model of the studied system was considered (Fig. 4) in order to make it easier to analyze how the system responds to changes over time. A resistor *R* with a small signal PV voltage represents the solar array *V̂pv* and current *I ^pv* throughout its terminals, while battery storage represents the load *R*_*O*_. By ignoring the battery load, the transfer function between the duty cycle *d ^(s)* as an input and the array voltage *V ^pv(s)* as an output of the system can be described as following^72^:

Where *f (D)* is the correlation between a boost converter's voltage at the output array *V*_*o*_ and the operating duty cycle *D*.

6 $${{\text{f}}\left({\text{D}}\right)={\text{V}}}_{{\text{PV}}}-\left(1-{\text{d}}\right){{\text{V}}}_{0}$$

The first derivation of Eq. (6) can be expressed by:

7 $$\dot{{\text{f}}}\left({\text{D}}\right)=-{{\text{V}}}_{0},$$

By inserting Eq. (7) into Eq. (5), we can obtain:

8 $$\frac{{{\text{V}}_{{{\text{pv}}}} \left( {\text{s}} \right)}}{{{\text{d}}\left( {\text{s}} \right)}} = \frac{{ - {\text{V}}/}}{{{\text{s}}^{2} + \frac{{{\text{L}}1}}{{{\text{RC}}_{1} }}{\text{s}} + \frac{1}{{{\text{LC}}_{1} }}}}$$

**Figure 5:**
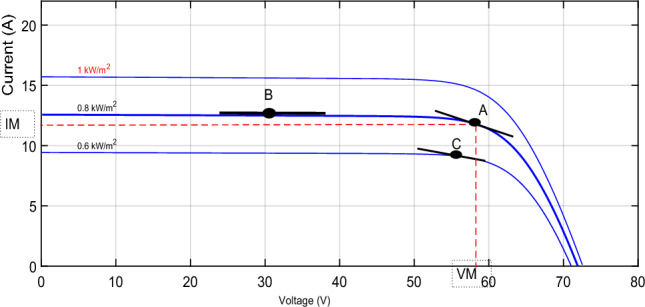
I-V curve of a solar array while changing R.

The negative sign in (8) implies that reducing the duty cycle *d(s)* causes the array voltage to increase. The transfer function depicted previously is derived from a linearized form^73^. Noteworthy is that *C*_1_ and *L* are identified, while *R* is unknown. The PV system's operating point (particularly the parameter *R*) fluctuates as ambient circumstances change quickly^74,75^. The value of *R* may be estimated by calculating the slope of the line tangent to point A on the I–V curve shown in Fig. 5 and then taking the inverse of that slope.

9 $$\frac{1}{{\text{R}}}=-\frac{\mathrm{\Delta I}}{\mathrm{\Delta V}} ,$$

## Proposed MPPT

**Figure 6:**
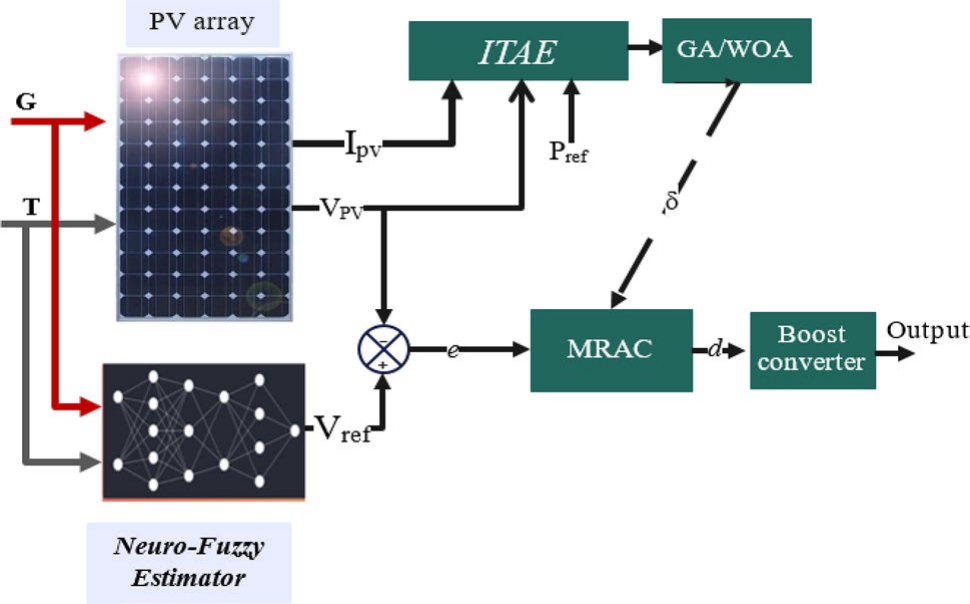
Block diagram of the suggested MPPT technique.

The proposed MPPT technique in this paper can be divided into three main steps, as depicted in Fig. 6. The first objective is maintaining a constant reference voltage (*V*_*ref*_) from the PV module output using an adaptive neuro-fuzzy controller under fluctuating environmental circumstances.

The ANFIS controller employs a relationship between VMPP and (irradiance and temperature) to supply reference peak power voltage. This reference value is compared with the value *V*_*pv*_ to provide an error value sent to the proposed MPPT's second stage. During the second control stage, the MRAC algorithm generates a control input that adjusts the DC–DC converter’s PWM signal. This paper also optimizes this controller using meta-heuristic optimization algorithms to ensure the most efficient dynamic performance of the system.

### Reference voltage generation via adaptive neuro-fuzzy algorithm (ANFIS)

To guarantee maximum power from the PV panel to the load, the MPPT controller must constantly check the PV array output voltage. Several methods have been used for calculating or learning the PV array reference peak power voltage. For instance, artificial neural networks (ANN)^60^, regression plans^49,76^, Gaussian process regression (GPR)^11^, and adaptive neuro-fuzzy inference systems (ANFIS)^39^. This paper uses the Neuro-Fuzzy algorithm to generate/estimate the voltage reference VMPP for the proposed controller.

**Figure 7:**
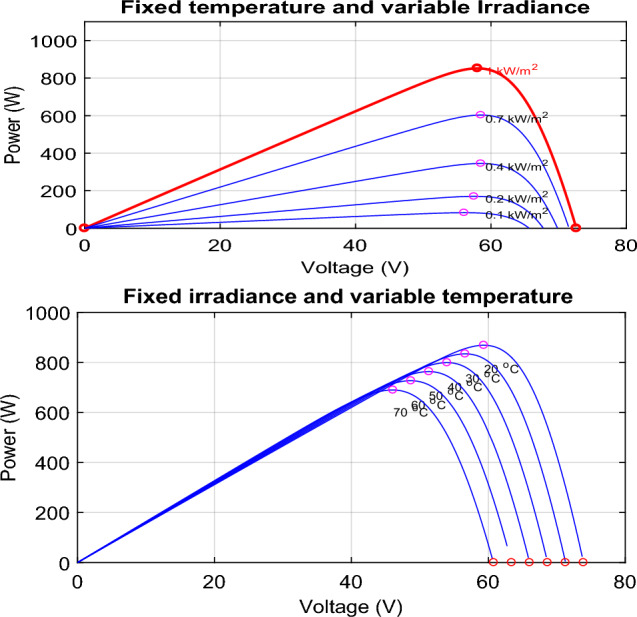
The characteristic curve of a photovoltaic module under varying environmental conditions.

**Figure 8:**
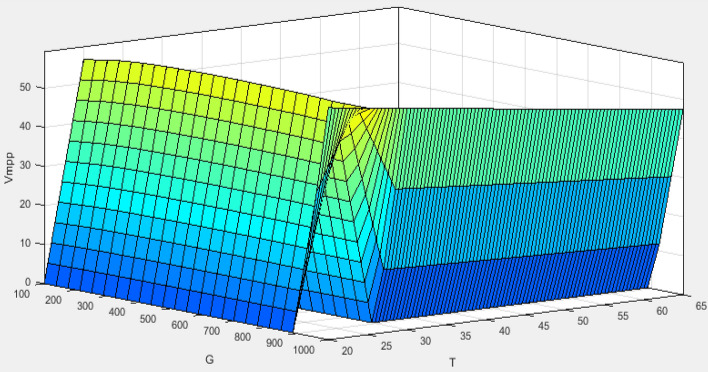
3D-plane.

**Figure 9:**
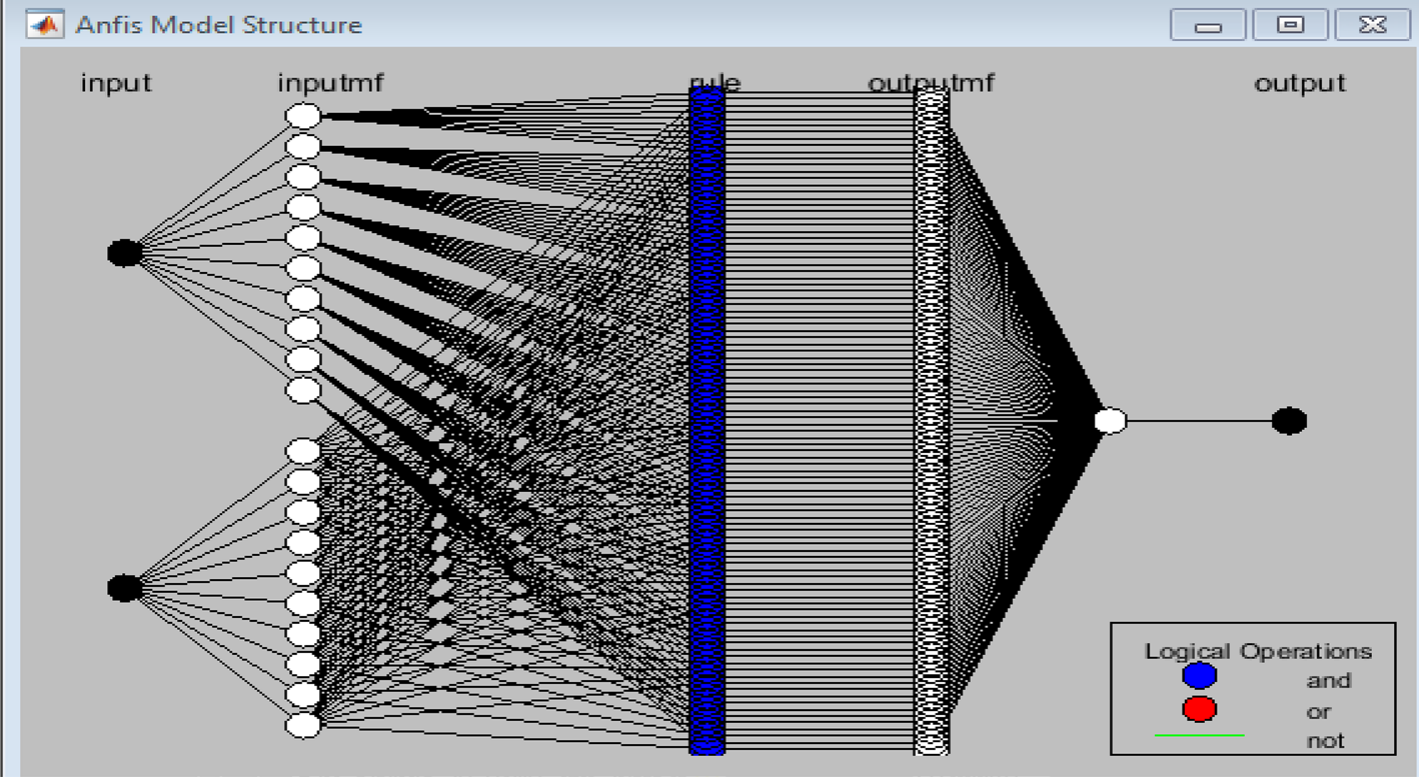
The architecture of NeuroFuzzy.

The curve of PV characteristic change under varying environmental conditions for every single temperature value (*T*) and irradiance (*G*) is presented in Fig. 7. The V_MPP_ data was collected using MATLAB/Simulink by adjusting the temperature from 20 to 70 °C while keeping the irradiance constant at 1000 w/m^2^. Then, we set the temperature to 25°, whereas the irradiance has been varied from 100 to 1000 w/m^2^. Figure 8 presents the result of these data as a 3-D plane. The structure of the ANFIS model in MATLAB/Simulink for VMPP estimation is depicted in Fig. 9. The neuro-fuzzy estimator comprises temperature (*T*) and irradiance (*G*) inputs. The fuzzification layer, which includes ten triangular membership functions assigned to every variable, represents each rule in the output layer as a linear equation^77,78^.

The ANFIS algorithm provides the reference voltage to the development controller. Then, the controller forces the output voltage to track the desired voltage to reach the maximum power point.

### Model reference adaptive control (MRAC)

An adaptive controller is, intuitively, one that can adapt its actions based on variations in the process dynamics and the nature of the disturbance^79^. Several researchers have attempted to define adaptive control in the literature. As shown in Fig. 9, we will use the pragmatic approach in this research, which involves a controller with adjustable parameters and a mechanism that allows these parameters to be adjusted. MRAC systems are designed so that the plant model's output constantly tracks the reference model’s output^80,81^. The structure of the model consists of a PV system with a boost converter, represented by a second-order transfer function, as shown in Eq. (8). The voltage reference V_ref_ produced by the first level (ANFIS estimator) is regarded as an input to the system, represented as *u *(*t*)*.* In contrast, *y *(*t*) represents the output. The plant model can be rewritten in the following manner:

10 $${{\text{G}}}_{{\text{p}}}\left({\text{s}}\right)=\frac{{\text{Y}}\left({\text{s}}\right)}{{\text{U}}\left({\text{s}}\right)}=\frac{{{\text{K}}}_{{\text{p}}}}{{{\text{s}}}^{2}+{{\text{a}}}_{{\text{p}}}{\text{s}}+{{\text{b}}}_{{\text{p}}}}$$

where $${{\text{k}}}_{{\text{p}}}=\frac{-{{\text{V}}}_{0}}{{{\text{LC}}}_{1}}, {{\text{a}}}_{{\text{p}}}=\frac{1}{{{\text{RC}}}_{1}}\mathrm{ and }{{\text{b}}}_{{\text{p}}}=\frac{1}{{{\text{LC}}}_{1}}$$

**Figure 10:**
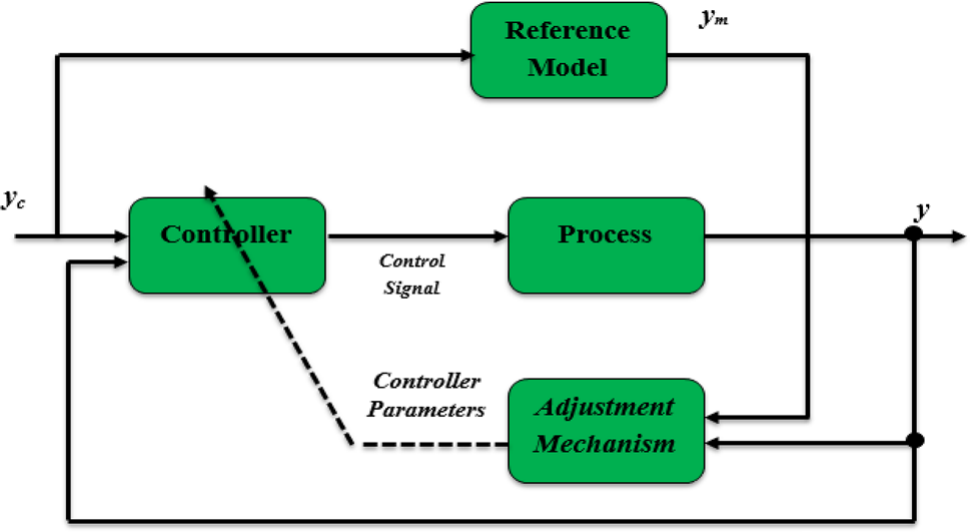
The architecture of the model references adaptive control.

The reference model that guides how the process output should ideally react to the control signal *u*(*t*) is given in Eq. (11). Where *y*_*m*_* is* the desired output, *K*_*m*_ represents a positive gain, and *a*_*m*_ and *b*_*m*_ must be selected. The reference model thus offers the suggested solution.

11 $${{\text{G}}}_{{\text{m}}}\left({\text{s}}\right)=\frac{{{\text{Y}}}_{{\text{m}}}\left({\text{s}}\right)}{{{\text{U}}}_{{\text{c}}}\left({\text{s}}\right)}=\frac{{{\text{K}}}_{{\text{m}}}}{{{\text{s}}}^{2}+{{\text{a}}}_{{\text{m}}}{\text{s}}+{{\text{b}}}_{{\text{m}}}}$$

The controller architecture depicted in Fig. 10 illustrates the approach we will use to achieve the control objective. The control law is described as follows^58^:

12 $${{\text{U}}={\uptheta }_{1}{\text{Y}}}_{{\text{c}}}-{\uptheta }_{2}{\text{Y}}={\uptheta }^{{\text{T}}}{\text{w}}$$

where *θ* = [*θ*_1_, *θ*_2_] are the parameters of the controller vector, and *ω* is described as *Y* = [*Y*_*c*_, *Y*]^*T*^.

**Figure 11:**
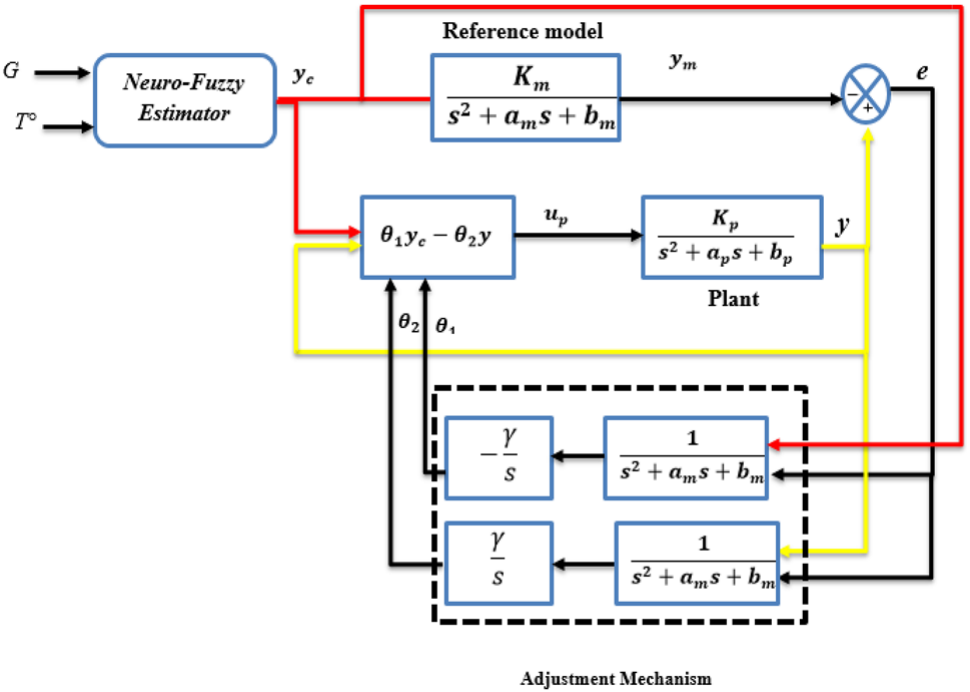
Structure of the controller in the proposed OMRAC.

In order to design the adaptation mechanism, MRAC theory uses two basic mathematical techniques. The MIT rule (Massachusetts Institute of Technology) is known as the gradient approach and Lyapunov stability theory. This study has chosen the MIT rule to adjust the controller’s parameters as an adaptation mechanism. By using the MIT rule, the loss function is expressed by:

13 $${\text{J}}\left(\uptheta \right)=\frac{1}{2}{{\text{e}}}^{2}$$

where e represents the error obtained by subtracting the plant y output value from the reference model output *Y*_*m.*_

14 $${\text{e}}={\text{Y}}-{\mathrm{Y }}_{{\text{m}}}$$

A mathematical equation was developed for updating the parameter *θ*, based on the assumption that, if the purpose were to minimize the error-related cost, moving towards the negative gradient of *J*. where *J* is supposed to change proportionally to *θ* would be advisable. Therefore, the derivative of *θ* equals the negative variation in *J*. Based on the cost function selected above, the following result can be obtained:

15 $$\frac{\partial\uptheta }{\partial {\text{t}}}=-\Upsilon \frac{\partial {\text{J}}}{\partial\uptheta }- {\Upsilon }_{{\text{e}}}\frac{\partial {\text{e}}}{\partial\uptheta }$$

Γ and *∂e/∂θ* are the system’s adaptation gain and sensitivity derivative, respectively.

The transfer function between the input *Y*_*C*_ and the plant’s output y is given by:

16 $$\frac{{\text{Y}}}{{{\text{Y}}}_{{\text{c}}}}=\frac{{{\text{K}}}_{{\text{p}}}{\uptheta }_{1}}{{{\text{s}}}^{2}+{{\text{a}}}_{{\text{p}}}{\text{s}}+\left({{\text{b}}}_{{\text{p}}}+{{\text{k}}}_{{\text{p}}}{\uptheta }_{2}\right)}$$

Thus, Eq. (14) can be rewritten as follows:

17 $${\text{e}}=\left[\frac{{{\text{K}}}_{{\text{p}}}{\uptheta }_{1}}{{{\text{s}}}^{2}+{{\text{a}}}_{{\text{p}}}{\text{s}}+\left({{\text{b}}}_{{\text{p}}}+{{\text{k}}}_{{\text{p}}}{\uptheta }_{2}\right)}-\frac{{{\text{K}}}_{{\text{m}}}}{{{\text{s}}}^{2}+{{\text{a}}}_{{\text{m}}}{\text{s}}+{{\text{b}}}_{{\text{m}}}} \right]{\mathrm{Y }}_{{\text{c}}}$$

According to (15), the sensitivity derivatives *∂e/∂θ*_*1*_ and *∂e/∂θ*_*2*_ can be described by:

18 $$\left\{\begin{array}{c}\frac{\partial {\text{e}}}{\partial {\uptheta }_{1}}=\frac{{{\text{K}}}_{{\text{p}}}}{{{\text{s}}}^{2}+{{\text{a}}}_{{\text{p}}}{\text{s}}+\left({{\text{b}}}_{{\text{p}}}+{{\text{k}}}_{{\text{p}}}{\uptheta }_{2}\right)}{{\text{Y}}}_{{\text{c}}} \\ \frac{\partial {\text{e}}}{\partial {\uptheta }_{2}}=\frac{{{\text{K}}}_{{\text{p}}}}{{{\text{s}}}^{2}+{{\text{a}}}_{{\text{p}}}{\text{s}}+\left({{\text{b}}}_{{\text{p}}}+{{\text{k}}}_{{\text{p}}}{\uptheta }_{2}\right)}Y\\ \end{array}\right.$$

In order to ensure a perfect tracking error from this close loop process, we will assume that the time behavior is the same as that of the reference model, as follows:

19 $${{\text{s}}}^{2}+{{\text{a}}}_{{\text{p}}}{\text{s}}+\left({{\text{b}}}_{{\text{p}}}+{{\text{k}}}_{{\text{p}}}{\uptheta }_{2}\right)={{\text{s}}}^{2}+{{\text{a}}}_{{\text{m}}}{\text{s}}+{{\text{b}}}_{{\text{m}}}$$

Therefore, based on the MIT rule, the control parameters are updated as follows:

20 $$\left\{\begin{array}{c}\frac{\partial {\uptheta }_{1}}{\partial {\text{t}}}=-\Upsilon \left[\frac{{{\text{K}}}_{{\text{p}}}}{{{\text{s}}}^{2}+{{\text{a}}}_{{\text{p}}}{\text{s}}+\left({{\text{b}}}_{{\text{p}}}+{{\text{k}}}_{{\text{p}}}{\uptheta }_{2}\right)}\right]e\left({\text{t}}\right) \\ \frac{\partial {\uptheta }_{2}}{\partial {\text{t}}}=-\Upsilon \left[\frac{1}{{{\text{s}}}^{2}+{{\text{a}}}_{{\text{m}}}{\text{s}}+{{\text{b}}}_{{\text{m}}}}\right]e\left({\text{t}}\right)\\ \end{array}\right.$$

The next step will be to tune the adaptation gain of the MRAC controller by employing two optimization techniques.

### Optimization techniques

This section provides two optimizer algorithms, namely GA and WOA, to adjust the adaptation gains of the proposed MRAC structure. It is worth mentioning that both these methods are widely recognized and have been frequently employed for similar applications.

#### Genetic algorithm (GA)

GA is an algorithm that is based on the population genetics concept. Each solution represents a chromosome, while each parameter corresponds to a gene^79,82,83^. GA employs an objective (fitness) function to assess the fitness of every single population member. The approach of retaining the most efficient solutions in each generation and utilizing them to generate the following solutions renders this algorithm reliable and proficient in approximating the best possible solution for a given problem^84,85^. In order to update the population, three genetic processes (selection, crossover, and mutation) are applied after each chromosome has been evaluated through a cost function and assigned a fitness value^51^. The genetic algorithm (GA) uses a selection operator (Boltzmann selection, Tournament selection, Rank selection, etc.)^86^ to allocate probabilities to individuals based on their fitness values. It allows for the selection of individuals to create the next generation in proportion to their fitness values. Once individuals are chosen via a selection operator, they must be utilized to produce the new generation. The chromosomes from the male and female genes are merged to generate a novel chromosome; this operation in GA is called crossover^87,88^. Mutation operators maintain diversity by adding randomness. By using this operator, the GA algorithm avoids local solutions and prevents solutions from becoming similar^43,79,89^,^90^.

In this study, the cost function chosen to evaluate the result of each individual in the population is Integral Time Absolute Error (ITAE), where the error (*e'*) can be obtained by subtracting the measured power from the maximum power (denoted *P*_*ref*_), as described in (21):

21 $${\text{ITAE}}=\int {\text{t}}\left|{{\text{e}}}^{\mathrm{^{\prime}}}\right|\partial {\text{t}}$$

Figure 11 depicts the procedure for adjusting the parameters of the MRAC controller with GA, which can be outlined using a flowchart, as shown below:

**Figure 12:**
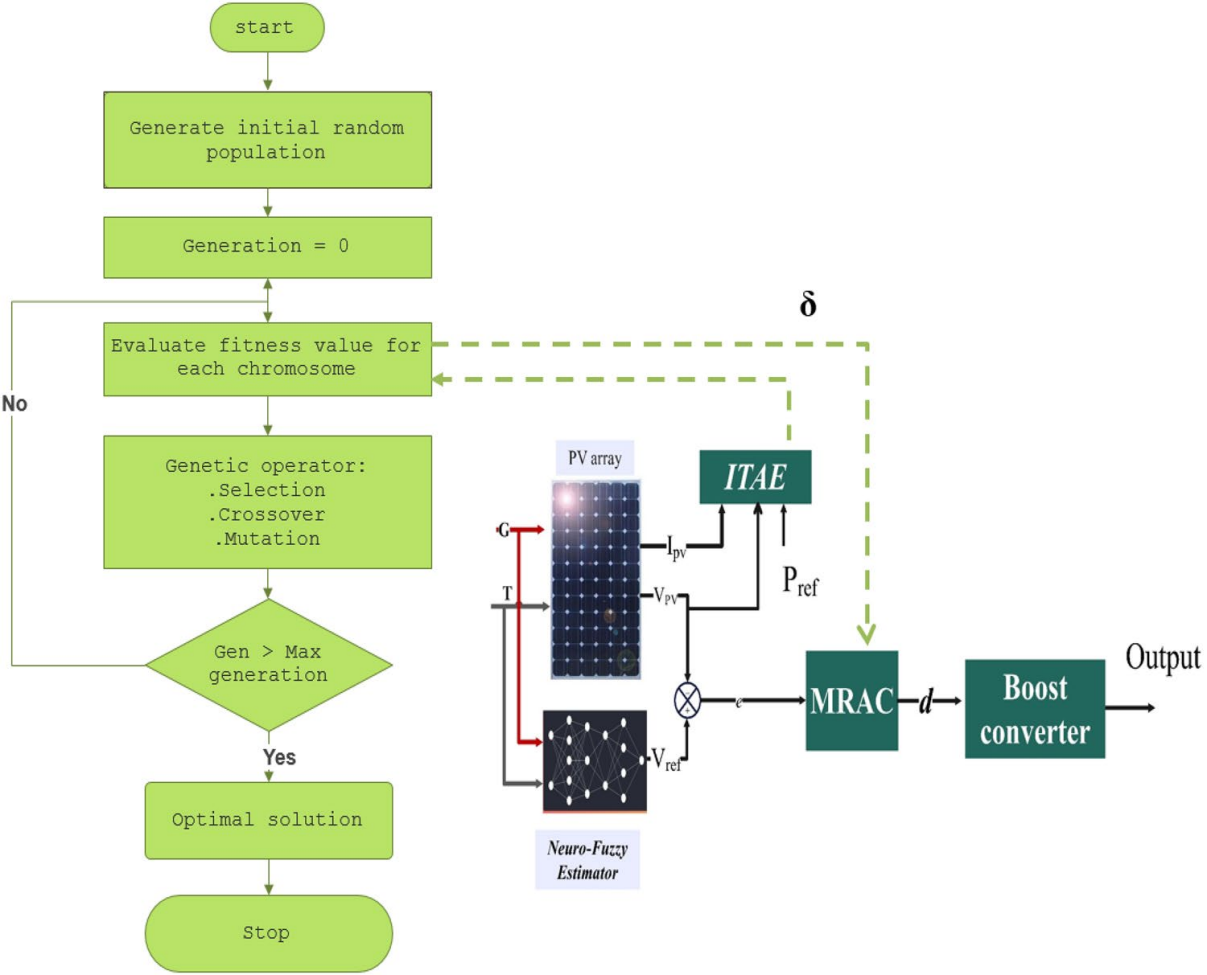
Block diagram for tuning of MRAC adaptive gain using GA.

#### Whale optimization algorithm

Highly intelligent and emotionally complex, whales have long captivated our imagination and inspired scientific research. Humpback whales are one of the most giant baleen whales. One of the most attractive aspects of humpback whales is their distinct hunting technique^73^. Based on this hunting technique, Mirjalili et al. introduced an innovative swarm intelligence algorithm called the Whale Optimization Algorithm (WOA)^59^. The humpback whale employs a distinctive hunting mechanism named the bubble net feeding method^91^. Noteworthy is that the bubble-net feeding method is a distinctive behavior exclusive to humpback whales. The hunting protocol of the humpback whale can be summarized in three steps:

*Encircling Prey*: Humpback whales can detect the whereabouts of their prey and surround them. In the Whale Optimization Algorithm (WOA), the precise location of the optimal design within the search space has yet to be discovered. Therefore, the algorithm postulates that the current foremost candidate solution is either the target prey or close to the optimal solution. Once the optimal search agent is identified, the remaining search agents adjust their positions toward the optimal search agent. This conduct is mathematically illustrated through the subsequent equations:

22 $$\overline{{\text{D}} }=\left|\overline{{{\text{X}} }^{*}}\left({\text{t}}\right).\overline{{\text{C}} }\overline{{\text{X}} }\left({\text{t}}\right)\right|$$

23 $$\overline{{\text{X}} }\left({\text{t}}+1\right)=\overline{{{\text{X}} }^{*}}\left({\text{t}}\right)-\overline{{\text{D}} }.\overline{{\text{A}} }$$

In the given equation, *t* denotes the present iteration, $\vec{C}$ and $\vec{A}$ serve as coefficient vectors, while X* represents a vector denoting the location of the best solution attained thus far. The process of determining the vectors $\vec{A}$ and $\vec{C}$ involves the following computations:

24 $$\overline{{\text{A}} }=2\overline{\mathrm{a }.} \overline{{\text{r}} }-\overline{{\text{a}} }$$

25 $$\overline{{\text{C}} }=2\overline{{\text{r}} }$$

The vector $$\vec{a}$$ gradually decreases linearly, starting by 2–0 over a series of iterations, whereas $$$$(\vec{r})$$$$ is a haphazard vector within the range [0,1].

*Attacking Mechanism Using Bubble-Net*: Two methods were developed to construct a mathematical representation for the bubble-net procedure of humpback whales: the Shrinking Encircling Mechanism and the Spiral Updating Position method^92^.

Shrinking encircling mechanism: This conduct can be achieved by decreasing the value of vector $\vec{a}$ in the Eq. (24). It is essential to highlight that vector $\vec{a}$ narrows the range of fluctuations in vector $\vec{A}$. Accordingly, the value of $\vec{A}$ inside the range [−*a*, *a*]*.* By assigning random values to $\vec{A}$ within the range of [− 1, 1], the search agent’s updated position can be between its original position and the current best agent’s position.

Spiral updating position: The method described in this approach involves computing the distance between a whale, which is positioned at coordinates (*X*, *Y*), and the location of its prey, which is located at coordinates (X*, Y*)*.* Hence, a spiral equation is formulated to represent the helix-shaped movement of the whales. This spiral equation is expressed as:

26 $${\overline{\text{X}}}\left( {{\text{t}} + 1} \right) = \overline{{{\text{X}}^{*} }} \left( {\text{t}} \right) + \left( {\overline{{{{{\rm D}^{\prime}}}}} .{\text{e}}^{{{\text{bt}}}} } \right){ }\cos \left( {2{\pi l}} \right)$$

While $\overline{{{{{\text D}^{\prime}}}}} = \left| {\overline{{{\text{X}}^{*} }} - {\overline{\text{X}}}} \right|$ represents how the whale is far away from the prey, where the prey is the best result achieved thus far, the constant b defines the logarithmic spiral’s form. At the same time, l serves as a stochastic number within the interval of [− 1, 1].

In order to simulate the whales’ coordinated movements, it is postulated that there is an even chance of 50% to opt for either the shortening encircling mechanism or the spiral model while the optimization procedure, to alter the whales’ positions simultaneously^20,56,93^. The mathematical representation of this model is given by:

27 $${\overline{\text{X}}}\left( {{\text{t}} + 1} \right) = \left\{ {\begin{array}{*{20}c} {\overline{{{\text{X}}^{*} }} \left( {\text{t}} \right) - {\overline{\text{A}}}.{\overline{\text{D}}}, \,\,\,\,\,\,\,\,\,\,\,\,\,\,\,\,\,\,\,\,\,\,\,\,\,\,\,\,\,\,\,\,if p \le 0.5} \\ {\overline{{{{{\text D}^{\prime}}}}} .{\text{e}}^{{{\text{bt}}}} \cos \left( {2{\pi l}} \right) + \overline{{{\text{X}}^{*} }} \left( {\text{t}} \right), \,\,\,\,if p \ge 0.5} \\ \end{array} } \right.$$

In the equation given, the variable *p* represents a random number within the range of [0 1].

*Exploration Phase*: During this phase, it is assumed that humpback whales search for prey randomly based on the positions of other whales. The model of this approach is expressed in this manner:

28 $$\overline{{{{{\text D}^{\prime}}}}} = \left| {{\overline{\text{C}}}.{\overline{\text{X}}}_{{{\text{rand}}}} - {\overline{\text{X}}}} \right|$$

29 $$\overline{{\text{X}} }\left({\text{t}}+1\right)={\overline{{\text{X}}} }_{{\text{rand}}}-\overline{\mathrm{A }.}\overline{{\text{D}} }$$

In the given equation, $\overrightarrow{X}{}_{rand}$ represents a whale’s haphazardly selected location data from the current iteration.

**Figure 13:**
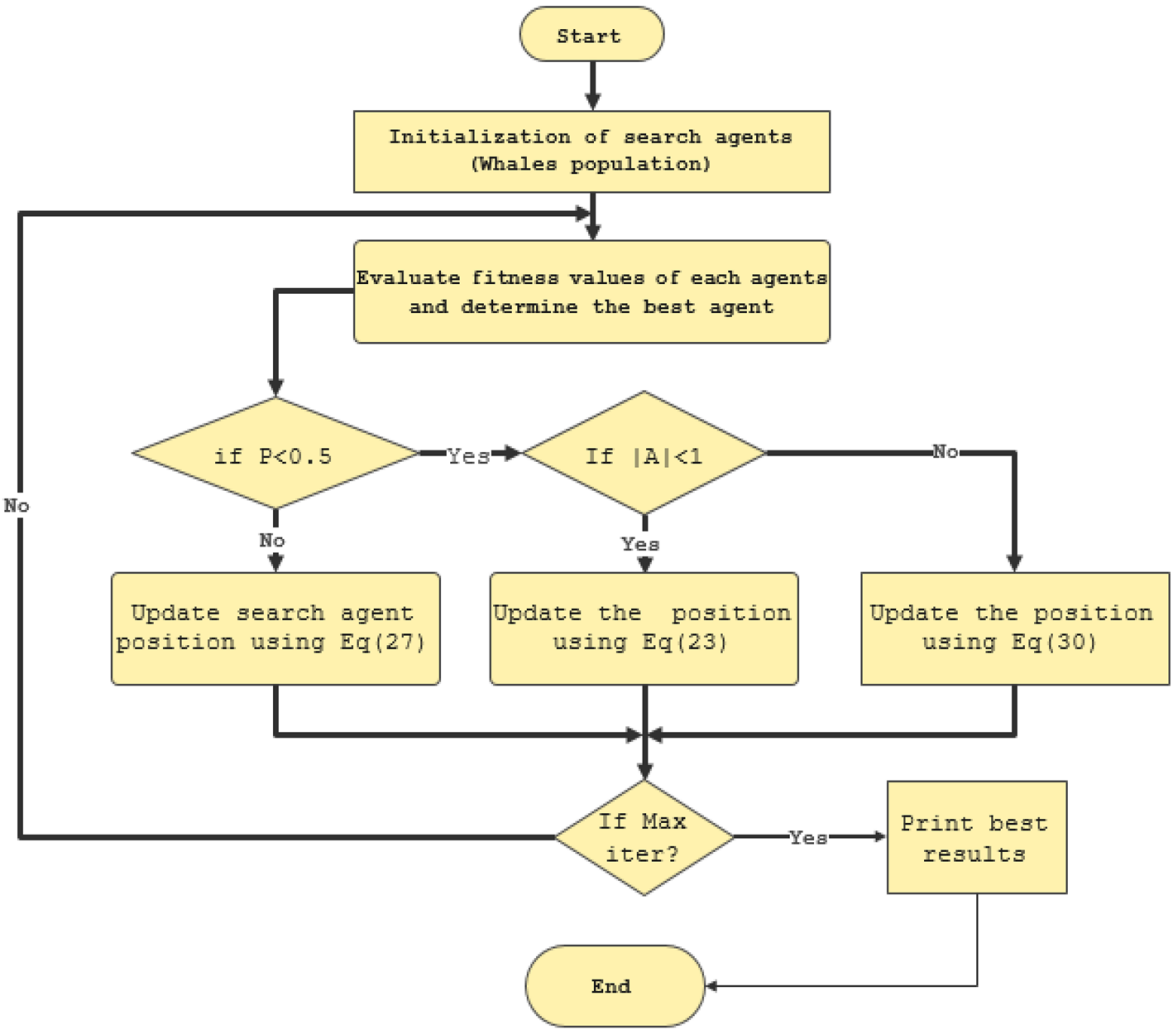
Whale optimization algorithm for tuning MRAC controller.

Using the same fitness function mentioned in (21), Fig. 13 illustrates the flowchart of the WOA.

## Simulation results and discussion

**Figure 14:**
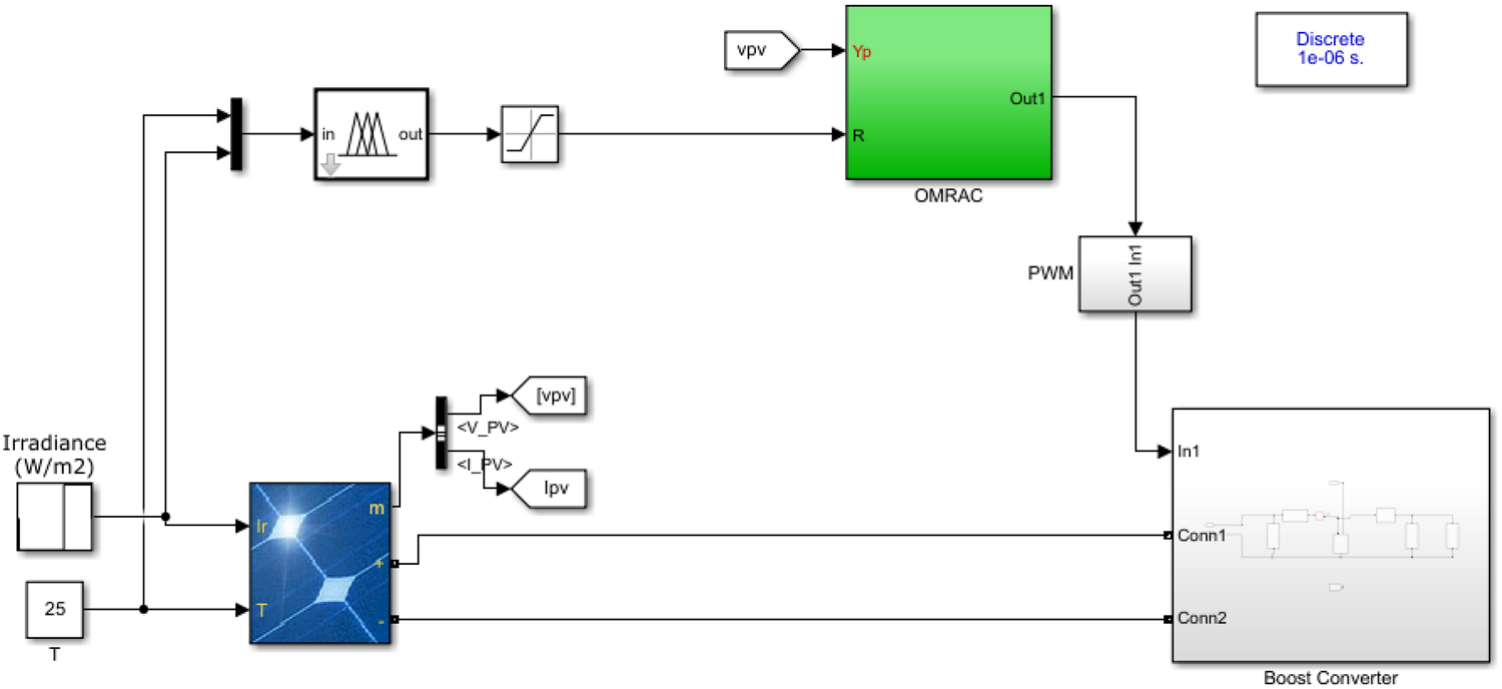
Implementation of the proposed model using MATLAB/SIMULINK environment.

**Figure 15:**
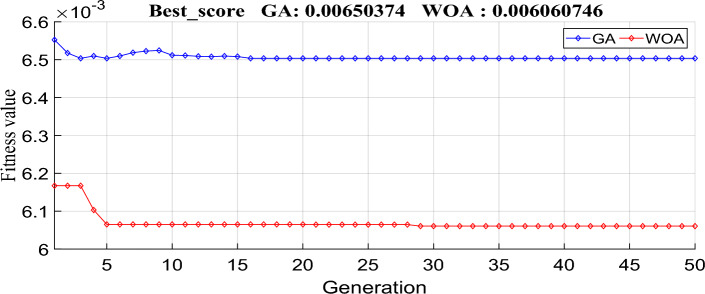
The performance of the objective function during the computation using GA and WOA.

To check the effectiveness of the developed ANFIS-OMRAC algorithm-based MPPT, this section was dedicated to simulating and analyzing the entire model system with Simulink, as shown in Fig. 14. The photovoltaic solar panel chosen in this work is the Soltech 1STH-215-P type. PV specifications and the DC–DC boost converter parameters are listed in Table 1. Indeed, GA and WOA are used to tune the MRAC controller design parameters. GA and WOA require fewer input parameters, which is one of the most significant advantages of these algorithms. The specifications for each algorithmic parameter are defined in Table 2. The number of search agents (population size) is relatively small considering the real-time implementation so that the controller may be optimized as quickly as possible. Figure 15 illustrates the objective function performance during computation. The whale optimization algorithm converges faster than the genetic algorithm. The whale optimization algorithm was more effective than the genetic algorithm concerning the convergence rate and accuracy of results. The optimum MRAC adaptation gain appears in Table 3.

**Table 1:** PV system characteristics.

Parameters	Values
The Peak Power of the PV panel (W)	213.15
Open circuit voltage V_OC_ (V)	36.3
Short circuit current I_SC_ (A)	7.84
The voltage corresponding to the maximum power V_MP_ (V)	29
The current corresponding to the maximum power I_MP_ (A)	7.35
Parallel strings (N_P_)	2
Number of cells associated in Series (N_S_)	2
Input capacitor ${{\text{C}}}_{1}$	100 µF
Output capacitor ${{\text{C}}}_{2}$	100 µF
Inductor L	2 mH
Restore load R	25Ω
Frequency f	20 kHz
Solver	1 e-6
Sample time	Ode 45

**Table 2:** GA and WOA parameters.

Genetic algorithm	
Population size	15
Crossover probability	0.8
Mutation function	Adaptive feasible
Population type	Double vector
Scaling function	Rank
Selection	Tournament
Maximum iteration	50

Whale optimization algorithm	
Population size	15
Maximum iteration	50
Cost function	ITAE

**Table 3:** The optimum MRAC controller gains.

Parameters		Values
Adaptation gain (γ)	0.161386	0.235809
Best score	0.006503	0.006074
Convergence	15th	5th iteration

After the optimal gain has been determined for OMRAC, the controller is used in online mode. Two different scenarios were used to evaluate the proposed MPPT technique. Firstly, under rapidly changing irradiation with a constant temperature. We will consider a uniform irradiance with a fast-changing temperature in the second test. On the other hand, a comparative study between the proposed GA-MRAC and WOA-MRAC, in addition to a conventional MPPT technique well known as incremental conductance (INC), is done in terms of the different indexes are: settling time (*ts*), Voltage ripple, Power ripple, the efficiency, integral absolute error (IAE) and integral square error (ISE). The efficiency is given by:

30 $${\text{Efficiency}}=\frac{{\int }_{{{\text{t}}}_{0}}^{{\text{t}}}{{\text{P}}}_{{\text{PV}}}\left({\text{t}}\right){\text{dt}}}{{\int }_{{{\text{t}}}_{0}}^{{\text{t}}}{{\text{P}}}_{{\text{MPP}}}\left({\text{t}}\right){\text{dt}}}$$

### Sudden change in irradiance level with a fixed temperature

**Figure 16:**
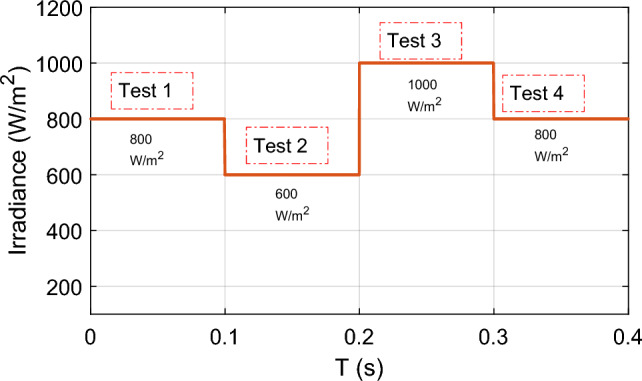
Solar irradiance profile.

**Figure 17:**
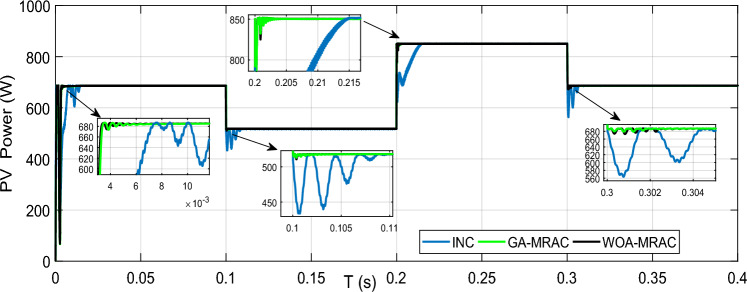
PV array power under variable irradiance for three MPPT techniques.

In this case, we consider the state of fast-changing irradiation levels, as shown in Fig. 16. We picked an irradiance profile with both step-up and step-down irradiation changes. In contrast, we set the temperature at the steady state, i.e., 25 °C. Figure 17 illustrates the PV power response for each technique used (GA-MRAC, WOA-MRAC, and INC). The MPPT performance depicted in Fig. 17 demonstrates that the settling time (TS) to reach MPP for the INC approach in the first test (Test 1) is 140 ms (ms). The GA-MRAC method takes 3.34 ms, while the WOA-MRAC method only requires 3.25 ms to reach MPP. The proposed optimal model reference adaptive controller (OMRAC) scheme exhibits superior dynamic performance using either GA or WOA. It quickly attains the MPP under irradiance variation and provides lower fluctuations around it throughout all four irradiation tests.

**Figure 18:**
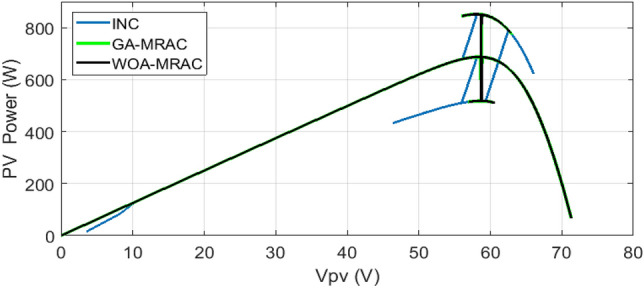
Power-Voltage characteristics under variable irradiation.

**Figure 19:**
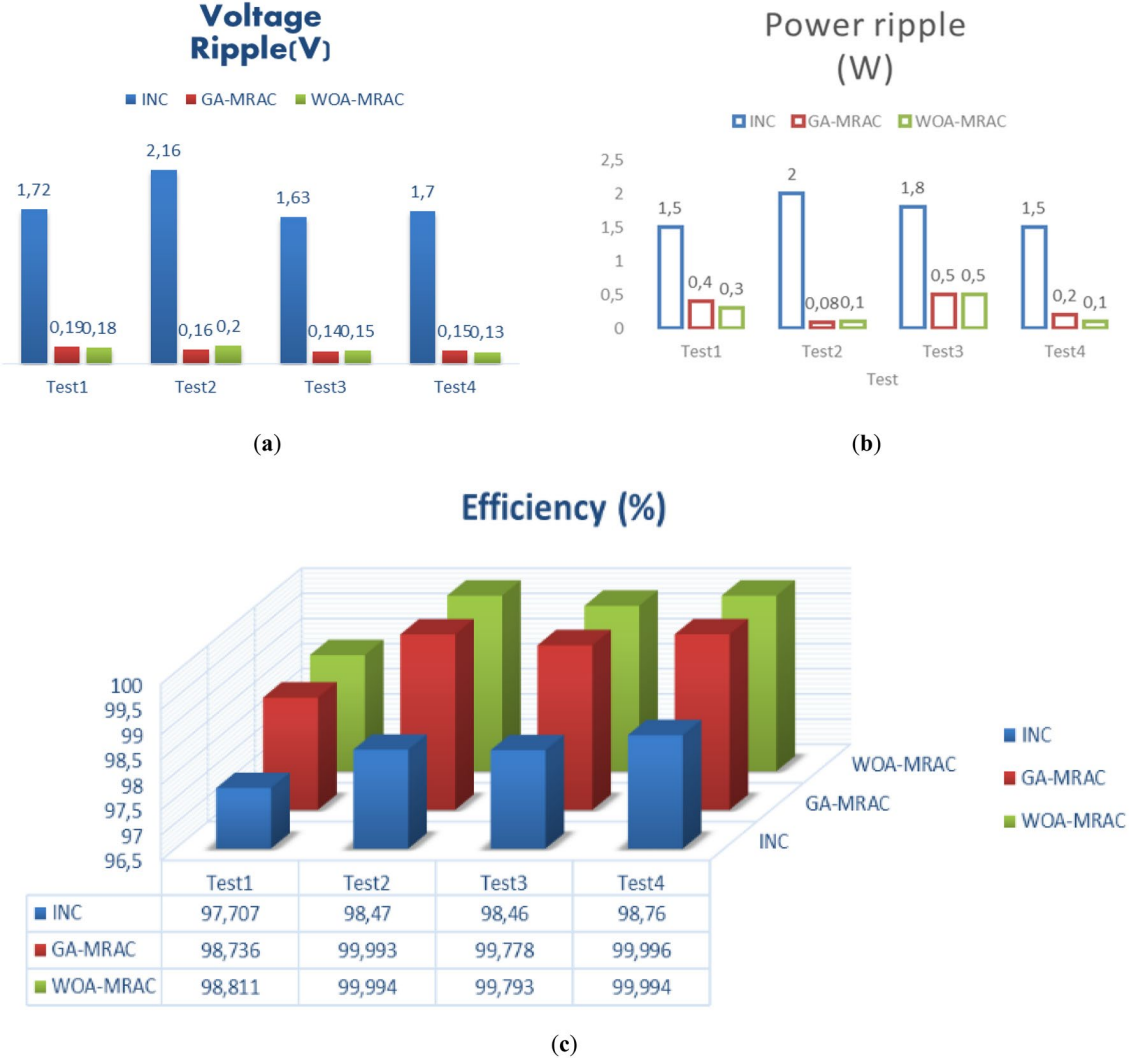
Comparison evaluation using the graphical representation: (**a**) Voltage ripple, (**b**) Power ripple, (**c**) Tracking efficiency.

The proposed OMRAC method provides better power quality than the INC method regarding power ripple. The suggested controller managed to decrease the oscillation around the MPP, as shown in Fig. 18, as well as low voltage fluctuation with an average efficiency of 99.92% for GA-MRAC and 99.65% for WOA-MRAC. Table 4 summarizes the dynamic performance results for each irradiation level applied in detail. Figure 19 illustrates the graphical representation of voltage, power ripple, and tracking efficiency.

**Table 4:** Analysis results for variable irradiation test.

Test	MPPT	Settling times (ms)	Voltage ripple (V)	Power ripple (W)	Efficiency (%)	Error (IAE)	Error (ISE)
1	INC	140	1.72	1.5	97.707	1.582	466.5
	GA-MRAC	3.34	0.19	0.4	98.736	0.868	362.4
	WOA-MRAC	3.25	0.18	0.3	98.811	0.8165	341.6
	INC	63.77	2.16	2	98.47	0.2653	11.2
2	GA-MRAC	0.547155	0.16	0.08	99.993	0.00576	0.04855
	WOA-MRAC	0.547155	0.2	0.1	99.994	0.00491	0.04897
	INC	12.6914	1.63	1.8	98.46	1.32	157.3
3	GA-MRAC	0.9169	0.14	0.5	99.778	0.1889	1.515
	WOA-MRAC	0.9533	0.15	0.5	99.793	0.1764	1.383
	INC	6.2046	1.7	1.5	98.76	0.2629	16.49
4	GA-MRAC	0.002589	0.15	0.2	99.996	0.00944	0.0618
	WOA-MRAC	0.8416977	0.13	0.1	99.994	0.01712	0.1151

### Under variable temperature and fixed irradiation

**Figure 20:**
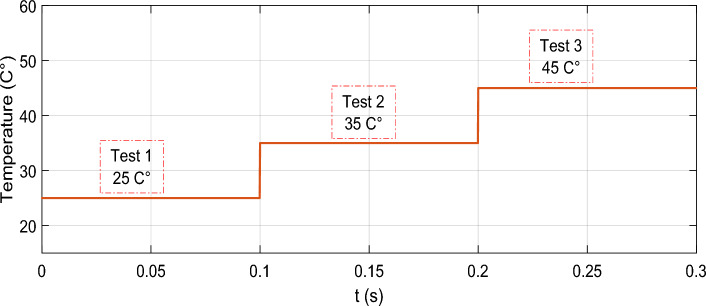
Temperature profile.

To carry out this case, the irradiation intensity was maintained at 1000 w/m^2^, while the temperature was manipulated by the temperature profile depicted in Fig. 20. A sudden shift of 10 °C was introduced to the temperature at each stage.

**Figure 21:**
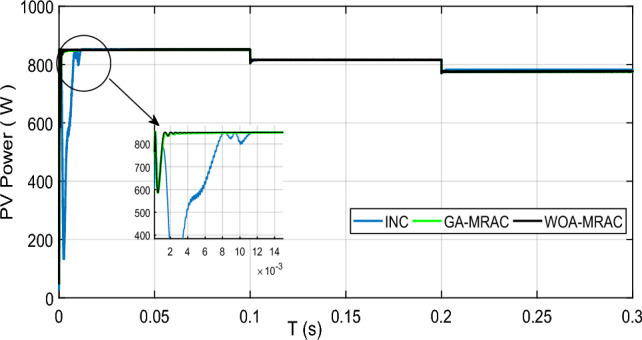
PV power output under variable temperature conditions.

**Figure 22:**
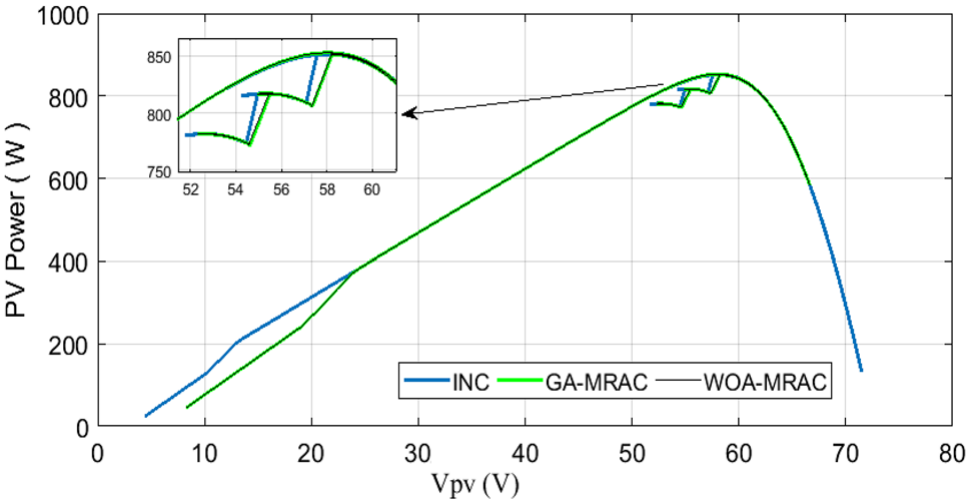
Power-Voltage curve under variable temperature.

Figure 21 shows the PV power output during a sudden temperature change using the proposed GA-MRAC and WOA-MRAC compared with the INC MPPT technique. It is worth mentioning that the suggested optimal MRAC exhibited significantly superior performance to INC. The traditional approach required 10.8 ms to monitor the maximum power point, whereas the GA-MRAC reaction time was calculated to be 1.8 ms. The WOA-MRAC MPPT converged in under 1.7 ms. Noteworthy, the OMRAC provides a superior improvement in convergence time under rapidly changing temperatures over all three temperature tests. The voltage *V*_*pv*_ generated by the photovoltaic (PV) array is depicted in Fig. 22; the optimal MRAC offers better *V*_*MPP*_ tracking with a small voltage ripple. On the other hand, there is more voltage fluctuation around *V*_*MPP*_ for the INC method.

**Figure 23:**
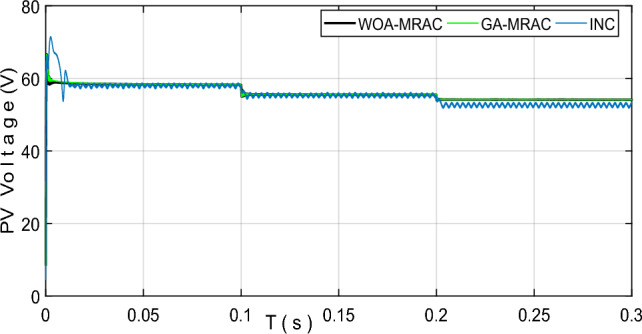
PV voltage output under variable temperature conditions.

Figure 23 shows the MPPT point tracking by three different algorithms. Accordingly, the suggested algorithm follows the MPP during varying temperature conditions with almost no power ripple.

**Figure 24:**
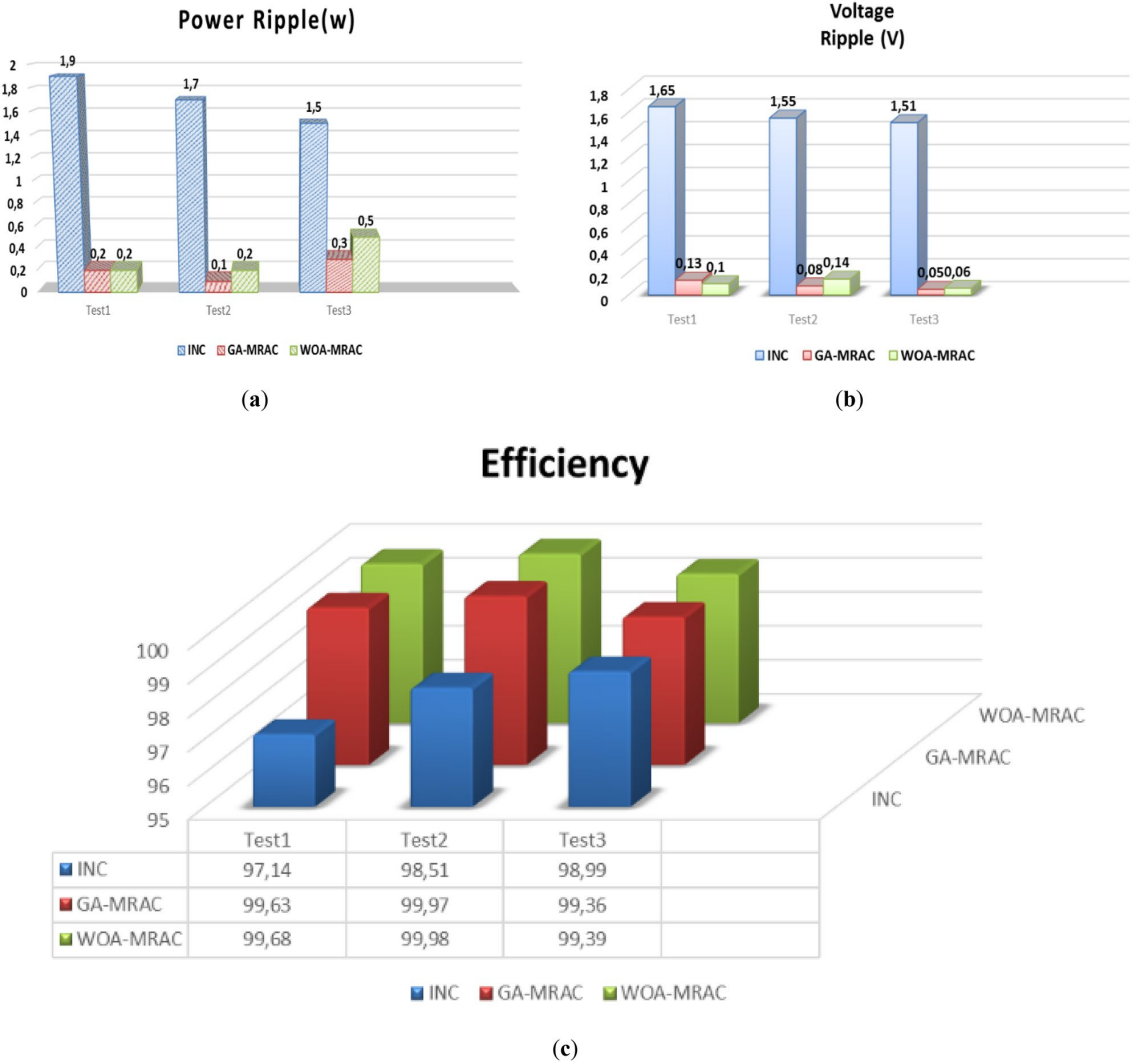
Comparison evaluation using the graphical representation under variable temperature: (**a**) Power ripple, (**b**) Voltage ripple, (**c**) Tracking efficiency.

Table 5 shows a comparative analysis of the mentioned techniques under various temperature tests regarding settling time (*T*_*s*_), Voltage ripple, power ripple, efficiency, IAE, and ISE. Addition to a graphical representation analysis to illustrate and support this comparative study is shown in Fig. 24. Through this table and graphic representation, we notice the superiority of WOA-MRAC, followed by GA-MRAC, and finally, the conventional INC technique. Table 6 thoroughly evaluates the most recent cutting-edge MPPT techniques.

**Table 5:** Analysis results for variable temperature test.

Test	MPPT	Settling times (ms)	Voltage ripple (V)	Power ripple (W)	Efficiency (%)	Error (IAE)	Error (ISE)
1	INC	10.895	1.65	1.9	97.14	2.435	989.8
	GA-MRAC	1.810545	0.13	0.2	99.63	0.3075	46.17
	WOA-MRAC	1.742495	0.1	0.2	99.68	0.2705	45.48
	INC	0.009627	1.55	1.7	98.51	0.04109	0.04575
2	GA-MRAC	0.000792	0.08	0.1	99.97	0.01873	0.01618
	WOA-MRAC	0.000799	0.14	0.2	99.98	0.0158	0.0196
	INC	0.0009611	1.51	1.5	98.99	0.04238	0.05594
3	GA-MRAC	0.000877	0.05	0.3	99.36	0.495	2.462
	WOA-MRAC	0.000891	0.06	0.5	99.39	0.474	2.258
	INC	10.895	1.65	1.9	97.14	2.435	989.8
4	GA-MRAC	1.810545	0.13	0.2	99.63	0.3075	46.17
	WOA-MRAC	1.742495	0.1	0.2	99.68	0.2705	45.48

**Table 6:** Comparison of the several MPPT methods found in the literature.

References	MPPT algorithms	Steady-state fluctuation	Tracking efficiency	Convergence	MPPT algorithms
^94^	Coarse and fine	Low	High	Low	Medium
^95^	IBA-FLC	Medium	High	Medium	High
^36^	ANN	Low	High	fast	Medium
^93^	Twisting Sliding Mode Control	Medium	Medium	fast	Low
^96^	Super twisting sliding mode-type two fuzzy	Low	Very high	fast	Low
^56^	P&O-MRAC	No	Very high	Very fast	Low
Proposed	ANFIS-WOA-MRAC	No	Very high	Very fast	medium

## Conclusions and future research directions

In this work, an innovative two-level maximum power point tracking (MPPT) controller designed for photovoltaic devices is developed. The first level of control includes the implementation of an Adaptive Neuro-Fuzzy (ANFIS) estimator to generate the voltage reference. To improve the MPPT algorithm efficiency even more, we introduced a second level of control using a model reference adaptive controller (MRAC). To optimize the MRAC controller performance, we employed two optimization techniques, Genetic Algorithm (GA) and Whale Optimization Algorithm (WOA), to fine-tune the controller's parameters. The proposed approach can cope with the challenges of quickly changing atmospheric conditions. The efficacy of the developed algorithm was verified through MATLAB/Simulink software. The performance of the innovative GA-MRAC and WOA-MRAC was compared with a conventional MPPT algorithm, namely incremental conductance (INC). Summing up the results, the WOA-MRAC controller exhibited the most excellent performance with the fastest convergence time, high tracking efficiency, and no further fluctuations. We conclude that the two-level MPPT control system shows promise for the optimization of PV systems, especially in dynamic environments where real-time tracking of the maximum power point is critical for energy harvesting.

In the future, several research directions could enhance the development of MPPT control systems for PV systems. Consider investigating alternative optimization algorithms other than genetic algorithms (GA) and whale optimization algorithms (WOA) to enhance the fine-tuning of MRAC parameters and maybe achieve superior outcomes. Evaluating the reliability of the two-level MPPT system in real-world scenarios, including partial shade, aging, and fluctuating load conditions, will offer vital insights into its practical use. Furthermore, combining the two-level MPPT system with additional control techniques like energy storage systems or grid-connected inverters could improve the system’s efficiency and reliability. Hardware prototypes and field tests are crucial for validating the performance of the two-level MPPT system in real-world situations, offering vital input for PV installations in both residential and commercial environments. Future study should prioritize optimizing and testing the two-level MPPT control system in real-world situations and investigating its integration with other control systems to improve performance.

## Data Availability

The datasets used and/or analysed during the current study available from the corresponding author on reasonable request.
